# Elucidating substituent effects in magnetic properties of redox active cobalt complexes and testing them as potential catalysts for HER†

**DOI:** 10.1039/d5ra01958c

**Published:** 2025-06-06

**Authors:** Sriram Sundaresan, Julia Müller, Luca M. Carrella, Eva Rentschler

**Affiliations:** a Department Chemie, Johannes-Gutenberg-Universität Mainz Duesbergweg 10–14 55128 Mainz Germany rentschler@uni-mainz.de ssundare@uni-mainz.de

## Abstract

We describe the synthesis of three cobalt complexes, C1–C3, featuring redox-active catechol ligands and a tetradentate salen ligand (L^sal^). Structural characterization using single-crystal X-ray diffraction at 120 K, along with UV-vis, infrared spectroscopy, and SQUID magnetometry, provided detailed insights into their electronic and geometric properties. Magnetic measurements revealed that the two tetrahalogenated catechol complexes (C1 and C2) remain diamagnetic up to 400 K, whereas the complex with 3,5-ditert butyl catechol (C3) shows radical characteristics and stays in semiquinone form strongly coupled with a Co(iii) centre. This radical behaviour was further confirmed by EPR spectroscopy in MeCN solution at room temperature. Cyclic voltammetry studies demonstrated the influence of catechol substituents on the electronic properties of these complexes, as reflected in shifts in oxidation potential. Preliminary investigations into their electrocatalytic activity for hydrogen production indicate that none of the complexes function effectively as catalysts under the tested conditions.

## Abstract 2

We report the synthesis of three cobalt complexes (C1–C3) with redox-active catechol ligands and a tetradentate salen ligand (L^sal^). Their magnetic properties were studied, and the complexes were also evaluated for electrocatalytic HER activity.

## Graphical abstract

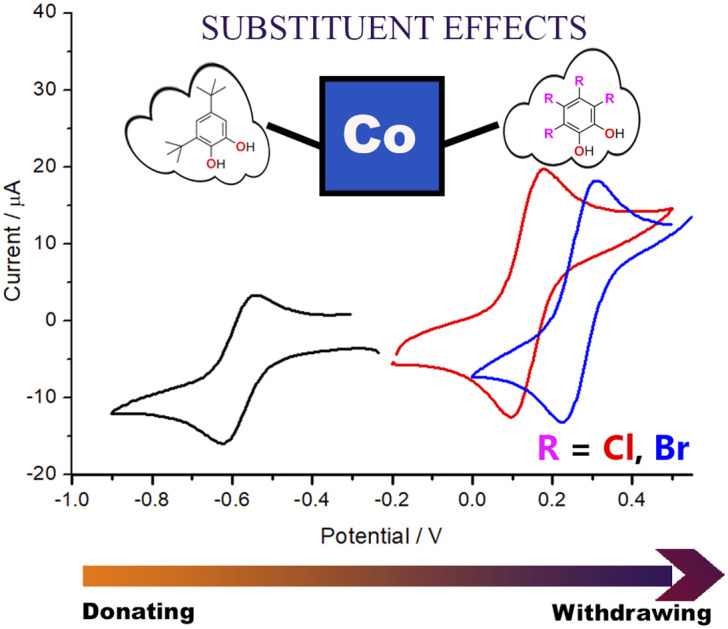

## 1. Introduction

The rising energy demand has driven research into alternatives to fossil fuels. Among renewable options such as solar, wind, and hydropower, hydrogen stands out for its zero emissions, high efficiency, and diverse production sources.^1–3^ Currently, 90% of global hydrogen is produced *via* steam reforming, generating significant greenhouse gas emissions and classified as brown hydrogen.^3–5^

A promising alternative is water splitting for hydrogen production *via* photo- or electrocatalysis, using catalysts to lower energy demands and overcome kinetic barriers.^2,4,6–8^ A promising alternative is water splitting for hydrogen production *via* photo- or electrocatalysis, using catalysts to lower energy demands and overcome kinetic barriers. While platinum-group metals excel in this process, their scarcity and high cost limit their viability for sustainable energy.^9,10^

Inspired by nature's ability to manage redox processes in metalloenzymes, researchers over the last decade have turned to 3d transition metals, in particular cobalt because of its readiness to access different oxidation states of +1, +2 and +3, to develop molecular catalysts for Hydrogen Evolution Reaction (HER). Due to their earth abundance, low cost, and tunable electronic properties, cobalt-based molecular systems have received considerable attention as HER catalysts. These complexes often contain nitrogen-coordinated ligands, such as porphyrins, salen and diglyoximes, which can be rationally designed to optimise catalytic performance.^11–13^.

Inclusion of redox-active ligands in cobalt complexes offers several advantages for electrocatalytic hydrogen production.^14,15^ These non-innocent ligands can act as electron reservoirs, facilitate multi-electron processes and potentially lower the overpotentials of the catalyst.^15,16^ In the current work, we report three cobalt complexes with catechol-based redox active ligands. The effect of the substituent in the catechol in tuning the magnetic and redox potentials is investigated in detail, along with preliminary testing for electrocatalytic HER.

## 2. Materials and methods

### 2.1 Experimental

All chemicals including the three catechol ligands were purchased from Alfa Aesar, Deutero, Fisher Chemicals, TCI, Sigma-Aldrich, and Acros Organics and used without further purification. Solvents were dried according to the literature-known procedures and used freshly distilled.^17^ NMR spectra were recorded at room temperature with a Bruker Avance DSX 400 and analysed with the program MestReNova. Magnetic susceptibility measurements were performed on a Quantum Design SQUID magnetometer MPMSXL in a temperature range between 200 and 400 K with an applied field of 1 kOe. All elemental analyses (Elementar vario EL Cube: C, H, and N) were measured at the microanalytical laboratories of the Johannes Gutenberg University Mainz. X-ray diffraction data were collected with a STOE STADIVARI at Johannes Gutenberg University Mainz. The structures were solved with ShelXT^18^ and refined with ShelXL^19,20^ implemented in the program Olex2.^21^ The X-ray cif file data are deposited on the Cambridge CCDC database with identification numbers 2415160–2415162. [Co_2_(H_2_O)(piv)_4_(Hpiv)_4_] used as a precursor was prepared as reported in the literature.^22^ For cyclic voltammetry measurements a PGSTAT potentiostat with a TSC 1600 was used with an already built-in platinum counter electrode as a vessel wall from rhd instruments. The working electrode was a glassy carbon electrode, which was polished before every measurement using aluminium oxide polishing paste with grain sizes of 0.1 μm and 0.05 μm from Buehler for at least half an hour on a microfiber cloth in figures of 8's. The reference electrode was an Ag/AgNO_3_ electrode. Tetrabutylammonium hexafluorophosphate was used in a concentration of 0.1 M as the conducting salt. The EPR spectra were recorded both in the solid state and in solution using a BRUKER Magnettech ESR 5000 and a cryo cooler with liquid nitrogen. The data in the case of solution phase measurements were fitted using Easy Spin 6.0.6. The UV-vis measurements were measured using a JASCO V-770 in single-beam mode. A data interval of 0.2 nm with a scanning speed of 400 nm min^−1^ was used. The data was then plotted with *Origin V7.5*. Infrared spectra were recorded at room temperature in a range of 4000–450 cm^−1^ using a Bruker ALPHA II ATR-IR with the Software *OPUS* and plotted using *Origin V7.5*. HRes ESI mass spectra were recorded on *Agilent 6200 series TOF/6500 series G-TOF* (11.0.203.0) at Johannes Gutenberg-University Mainz in acetonitrile or methanol.

#### 2.1.1 Ligand synthesis

##### 2.1.1.1 Synthesis of L^sal^

The ligand was synthesised in accordance with the literature with modifications.^23,24^ Salicylaldehyde (0.244 g, 1.998 mmol, 2.002 eq.) and 1,3-diaminopropane (0.074 g, 0.998 mmol, 1.000 eq.) were dissolved in 20 mL of dry methanol. The bright yellow solution formed was stirred at room temperature for one day. The solvent was removed under reduced pressure and the product (L^sal^) was obtained as a yellow solid in a high yield (0.223 g, 0.950 mmol, 79%). FT-IR: *ν̃* [cm^−1^]: 3051, 2944, 2893, 1630, 1578, 1496, 1459, 1415, 1337, 1275, 1209, 1146, 1125, 1100, 1081, 1051, 1005, 970, 882, 853, 779, 748, 736, 661, 639, 571, 556, 521. 1H-NMR (DMSO-d_6_[400 MHz]): *δ* [ppm] = 13.52 (s, 2H, H^1^), 8.60 (s, 2H, H^4^), 7.44 (d, 2H, *J*^3^ = 8.0 Hz, H^9^), 7.34 (t, 2H, *J*^3^ = 8.0 Hz, H^8^), 6.90 (m, 4H, *J*^3^ = 6.7 Hz, H^6,7^), 3.69 (t, 4H, *J*^3^ = 6.0 Hz, H^2^), 2.03 (q, 2H, *J*^3^ = 7.0 Hz, H^1^). Elemental analysis: calculated: C 72.32%, H 6.43%, N 9.92%, found: C 72.12%, H 6.79%, N 10.20%.

314 nm (C1), 316 nm (C2), and 302 nm (C3)

#### 2.1.2 Complex synthesis

##### 2.1.2.1 (NH(Et)_3_)[Co^III^(tccat)(L^sal^)] (C1)

To a solution of [Co_2_(H_2_O)(piv)_4_(Hpiv)_4_] (0.081 g, 0.085 mmol, 1.000 eq.), L^sal^ (0.048 g, 0.170 mmol, 2.000 eq.) and tetrachlorocatechol (0.042 g, 0.170 mmol, 2.000 eq.) in 12 mL of acetonitrile triethylamine (0.034 g, 0.340 mmol, 4.000 eq.) was added. A dark green mixture formed. The mixture was stirred under reflux for one hour and was left to cool down to room temperature while stirring. When the mixture was at room temperature it was filtrated. The filtrate was dark green and single crystals suitable for X-ray data collection were formed after three days. The product obtained in moderate yields was filtered and air dried (0.037 g, 0.05 mmol, 32%). FT-IR: *ν̃* [cm^−1^]: 2984, 2933, 2661, 2352, 2248, 1625, 1595, 1538, 1442, 1397, 1366, 1338, 1309, 1292, 1254, 1227, 1211, 1197, 1147, 1127, 1084, 1065, 1031, 1008, 981, 968, 942, 898, 845, 793, 752, 736, 600, 583, 553, 532, 518, 498, 481, 460, 433, 409. Elemental analysis calculated C 50.68%, H 4.70%, N 6.11%, found: C 50.11%, H 4.92%, N 6.55%. UV-vis in MeCN: 314 nm, 398 nm.

##### 2.1.2.2 (NH(Et)_3_)[Co^III^(tbcat)(L^sal^)] (C2)

To a solution of [Co_2_(H_2_O)(piv)_4_(Hpiv)_4_] (0.062 g, 0.065 mmol, 1.000 eq.), L^sal^ (0.037 g, 0.130 mmol, 2.000 eq.) and tetrabromocatechol (0.055 g, 0.130 mmol, 2.000 eq.) in 12 mL of acetonitrile triethylamine (0.026 g, 0.260 mmol, 4.000 eq.) was added. A dark green mixture was obtained. The mixture was stirred under reflux for one hour and was allowed to cool down to room temperature with stirring. When the mixture was at room temperature it was filtrated. The filtrate was dark green, and single crystals suitable for X-ray diffraction were formed after three days. The product obtained in low yields was filtered and air dried (0.026 g, 0.03 mmol, 23%). FT-IR: *ν̃* [cm^−1^]: 2971, 2662, 1637, 1624, 1594, 1539, 1467, 1432, 1388, 1358, 1338, 1291, 1267, 1231, 1217, 1193, 1145, 1127, 1093, 1064, 1032, 1018, 971, 961, 949, 925, 900, 842, 799, 760, 749, 735, 646, 597, 568, 550, 518, 505, 483, 468, 447, 432, 421, 400. Elemental analysis calculated C 40.26%, H 3.73%, N 4.86%, found: C 40.22%, H 3.81%, N 4.70%. UV-vis MeCN: 316 nm, 398 nm.

##### 2.1.2.3 [Co^III^(3,5dbcat)(L^sal^)]·CH_3_CH_2_OH (C3)

To a solution of Co(ClO_4_)_2_·6H_2_O (0.066 g, 0.018 mmol, 1.000 eq.) in 2.5 mL of ethanol, a solution of L^sal^ (0.051 g, 0.181 mmol, 1.006 eq.), triethylamine (0.036 g, 0.356 mmol, 1.987 eq.) and 3,5-dbcat (0.040 g, 0.180 mmol, 1.000 eq.) in 3 mL of ethanol was added dropwise. The dark brown mixture was stirred at room temperature for 30 min and then filtrated. The product was obtained as brown crystals by slow evaporation after one day. Single crystals suitable for X-ray diffraction were grown after one day. The product obtained in low yields was filtered and air dried (0.030 g, 0.05 mmol, 28%) FT-IR: *ν̃* [cm^−1^]: 2950, 2867, 1625, 1595,1572, 1537, 1444, 1394, 1346, 1327, 1301, 1243, 1205, 1146, 1127, 1081, 1030, 985, 960, 899, 852, 827, 757, 749, 739, 695, 619, 599, 576, 558, 500, 466. Elemental analysis calculated, C, 65.50%, H, 6.98%, N, 4.85%; found, C 65.86% H 6.70%, N 5.15%. UV-vis in MeCN: 302 nm, 395 nm.

## 3. Results and discussion

### 3.1 Synthesis

The salen ligand was synthesized according to the procedure reported in the literature by a Schiff base condensation of salicylaldehyde and propylene diamine.^23,25^ The ligand was characterised using a range of techniques, including ^1^H-NMR and ^13^C-NMR spectroscopy. In the ^1^H-NMR spectrum, the appearance of an imine proton signal around 8.5 ppm, the absence of the aldehyde proton signal near 9.5 ppm, and the presence of a hydroxyl proton signal around 13.5 ppm confirm the successful formation of the ligand (Fig. S1 and S2†). High-resolution ESI-positive mass spectrometry further confirmed this, with a principal peak observed at *m*/*z* 283.145, consistent with the protonated L^sal^ ligand. Additionally, FTIR spectroscopy revealed a characteristic imine stretching vibration at 1630 cm^−1^ and a broad O–H stretching band around 3300 cm^−1^, while the absence of the aldehyde C=O stretching vibration near 1690 cm^−1^ indicated complete Schiff base condensation, confirming the formation of the ligand. The catechol ligands used for complexation in all three cases were purchased commercially.

The synthesized L^sal^ ligand was used for the complexation reactions with the appropriate catechol and metal salt (see Experimental section for details). The resulting complexes were characterised by single-crystal X-ray diffraction (SCXRD) at 120 K, using crystals harvested from the mother liquor. Bulk samples obtained following filtration and drying were characterised by elemental analysis. In all three cases, the lattice solvent, initially acetonitrile, was replaced by water, and the samples were isolated as monohydrates, consistent with observations reported in the literature.

High-resolution electrospray ionisation mass spectrometry in negative mode (HRESI-MS) confirmed the formation of the desired complexes, with the major peaks corresponding to the expected molecular ions and matching the calculated isotopic patterns (Fig. S7–S10†). Additional fragment ions, attributed to partial decomposition under the harsh ionisation conditions typical for labile 3d metal complexes, were also observed. It should be noted that mass spectrometry does not serve as a direct indicator of sample purity but is presented here as supplementary evidence of complex formation.

**Fig. 1:**
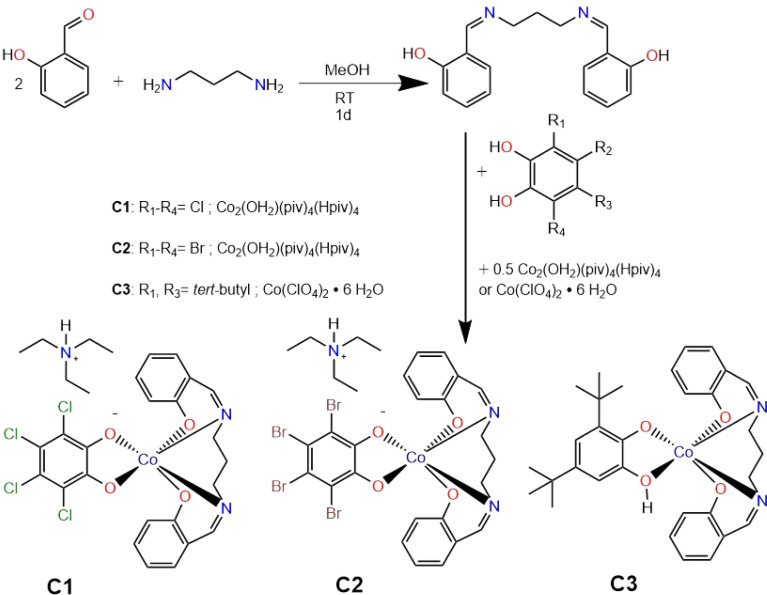
Synthesis of L^sal^ ligand and corresponding complexes C1–C3.

Further support for complexation was obtained from infrared spectroscopy (IR), where a shift in the imine (C=N) stretching frequency, along with the disappearance of the free phenolic O–H stretching vibration, was observed. Notably, for complexes C1 and C2, the strong electron-withdrawing effect of Co^3+^ in combination with the π-acceptor character of the catechol ligand strengthens the C=N bond, leading to a shift of the imine stretching band to higher wavenumbers. In contrast, in complex C3, the semiquinone ligand donates greater electron density to the cobalt centre, thereby reducing the effective positive charge on the metal and weakening the C=N bond. Consequently, the imine stretching frequency shifts to lower wavenumbers relative to the free ligand (Fig. S3–S6†). The ChemDraw sketches of all three complexes can be seen in Fig. 1.

### 3.2 Crystal structure description

**Fig. 2:**
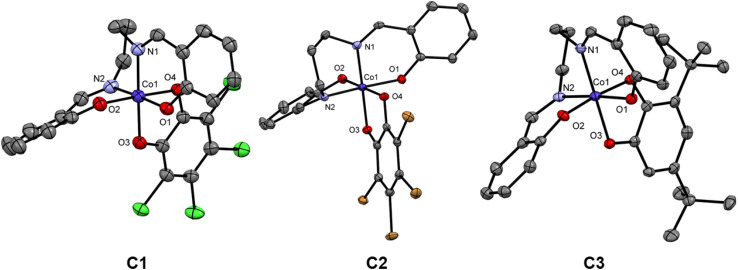
Crystal Structures of complexes C1–C3 at 120 K. Triethyl ammonium counterion as well as the hydrogen atoms are eliminated for clarity.

Complex C1 and C2 crystallized as brown, prismatic single crystals from acetonitrile *via* slow evaporation within three days. The complexes crystallized in a monoclinic crystal system in the space group *C*2/*c* (C1) and *P*2/*n* (C2), respectively. The X-ray data was collected at 120 K. The crystallography data are tabulated in Tables S1 and S2.† The asymmetric unit consists in both cases of the complex cation and one triethyl ammonium counterion. The packing is depicted in Fig. S16 and S17.† Complex C1 and C2 consist of one central cobalt atom coordinated by one tetradentate L^sal^, and the tetra halogenated (Cl-C1; Br-C2) catecholate (Fig. 2). The metal donor bond lengths can be found in Table S4.† The average bond lengths Co–O with 1.908/1.905 Å and Co–N with 1.912/1.908 Å, for C1 and C2, indicate that cobalt is in the oxidation state +III in a LS state.^11,12^

Complex C3 crystallized as brown, cuboid single crystals from ethanol by slow evaporation within one day. The complex crystallizes in a triclinic system in the space group *P*1̄ as a mononuclear cobalt complex. The X-ray data were collected at 120 K. For the crystallography data see Table S3.† The asymmetric unit comprises two complexes and one solvent molecule (Fig. S18†). C3 consists of the central cobalt atom, one tetradentate L^sal^ and the 3,5-dbcat ligand (Fig. 2). The metal donor bond lengths can be found in Table S4.† The average bond length of Co–O with 1.905 Å and Co–N with 1.915 Å indicate that cobalt again is in the oxidation state +III in a LS state.^11,12^ As Since there is no counterion and the ligand L^sal^ is doubly negatively charged in its deprotonated form, the dioxolene unit must be present in its semiquinoid radical form for a Co^III^ ion. The presence of a semiquinone is confirmed by the bond lengths within the 3.5-dbcat, as indicated by the localized double bond character within the aromatic ring (Table S5†). In addition to this, the innocent nature of the imine ligand from the L^sal^ is also verified from the average bond length of 1.287 Å (Table S6†) for all three complexes and also from the overall charge balance.

### 3.3 UV-vis

**Fig. 3:**
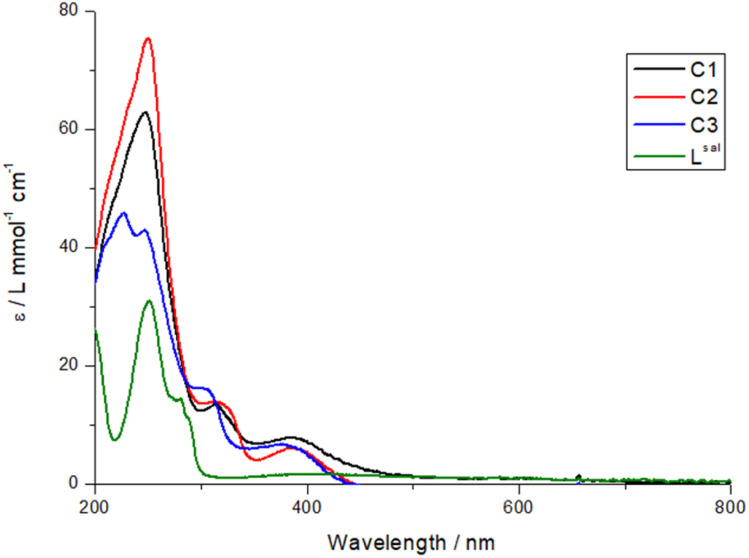
UV-vis spectra of complexes C1–C3 in acetonitrile at room temperature.

The UV-vis spectra of all three complexes, C1–C3, were measured in acetonitrile at room temperature at a concentration of 1 × 10^−4^ mol L^−1^ (Fig. 3). The UV-vis spectra of the complexes, when compared with the spectrum of L^sal^, clearly show that the maximum at 250 nm is attributable to a ligand-based transition. On the other hand, the absorption maxima at 314 nm (C1), 316 nm (C2), and 302 nm (C3) can be assigned to the catecholate intraligand π → π* transition.^26–28^ The observed blue shift for the 3,5-di-*tert*-butylcatechol cobalt complex C3 compared to the tetrahalogenated catechol cobalt complexes C1 and C2 is due to electronic effects. The bulky *tert*-butyl groups in di-*tert*-butylcatechol are electron-donating. Furthermore, in the case of the C3 complex, the catechol is in the semiquinoid state, which reduces π conjugation and increases the energy of the π ligand orbitals. This results in a transition at a higher energy. In contrast, halogen substituents (Cl or Br) in tetrahalogenated catechols have an electron-withdrawing effect, lowering the energy of the π orbitals and enhancing π donation to the cobalt center, resulting in a CT transition with a lower energy. In addition, the tetrahalogenated catechols coordinated to cobalt increase its electrophilicity and promote π-back bonding, further stabilizing the CT band in the tetrahalogenated complexes. These shifts are consistent with the literature.^26–28^

The band at about 400 nm can be assigned to the LMCT, as is commonly seen in the literature.^29–31^ In the case of the C3 complex, the monoanionic semiquinone introduces a delocalized unpaired electron that modifies the LMCT transition and causes a slight blue shift in the maxima. On the other hand, the electronic effects mediated by the tetrahalogenated catechols are quite similar, as evidenced by no shift in the LMCT bands.

### 3.4 Magnetic data analysis

**Fig. 4:**
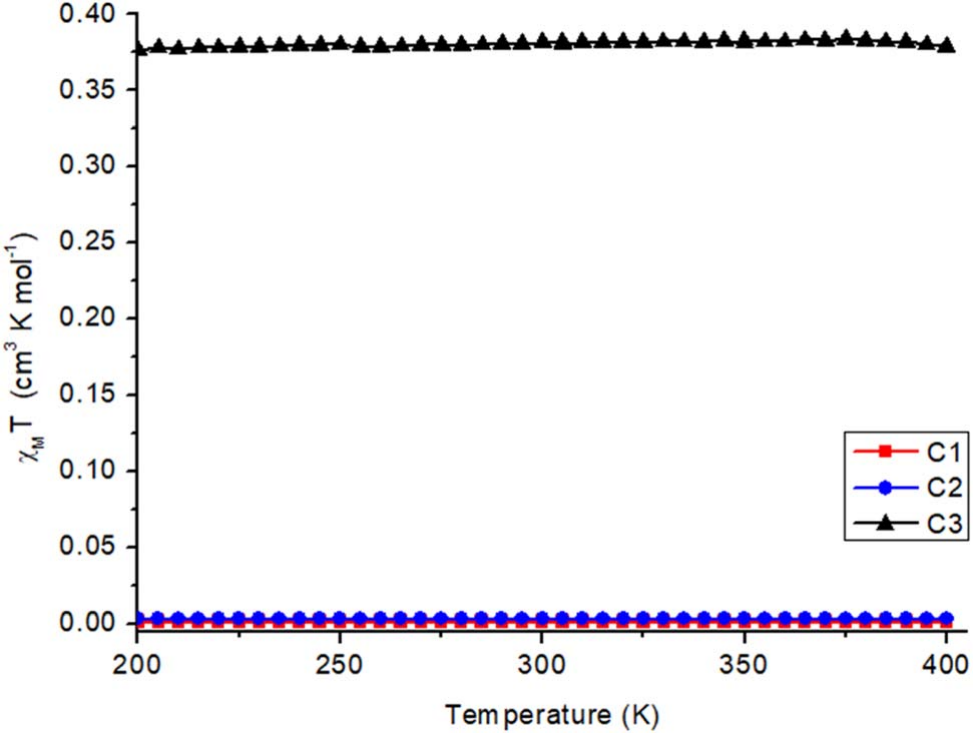
*χ*_M_ *T vs. T* for complexes C1–C3 between 200–400 K. The data points are triangles, dots, and squares for the complexes C1–C3, respectively, and the line is just the guide for the eye.

The magnetic data for C1 and C2, recorded from 400–200 K in an applied field of 1000 Oe (Fig. 4), revealed that these two complexes are diamagnetic throughout the measured temperature window. This was expected from the X-ray crystal structure analysis, indicating that the Co(iii) is in the LS *S* = 0 state and the catechols are in the catecholate form.

Complex C3 was measured from 400 K to 10 K (Fig. 4 and S15†). As expected from the structural results, a semiquinone radical coordinated to the diamagnetic Co(iii) (d^6^, *S* = 0) leads to a magnetic moment of 0.373 cm^3^ K mol^−1^ for the complex C3. This is in accordance with the literature.^14^

### 3.5 EPR spectroscopy

**Fig. 5:**
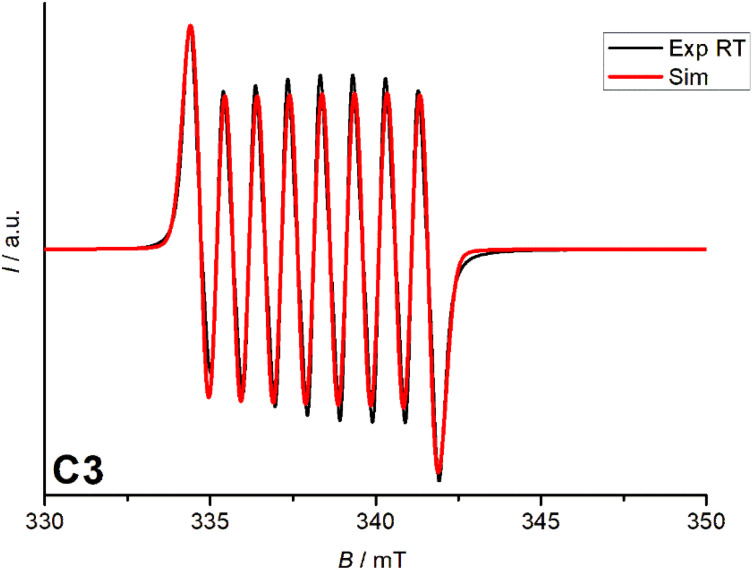
EPR spectrum of C3 in MeCN at room temperature in the range of 330–350 mT.

The EPR spectrum of the Co(iii)-semiquinone complex in MeCN at room temperature shows a coupling between the low-spin Co(iii) center (*S* = 0) and the semiquinone radical (*S* = 1/2). The hyperfine structure of the ^59^Co nucleus (*I* = 7/2) results in an eight-line pattern with a constant of 27 MHz, indicating limited spin density transfer. The *g*-value of 2.00, as expected for a free electron value (*g* = 2.00), suggests a ligand-based radical character. These features confirm the delocalization of an unpaired electron over the ligand and cobalt centre, which is characteristic of Co(iii)-semiquinone systems (Fig. 5).

### 3.6 Cyclic voltammetry

**Fig. 6:**
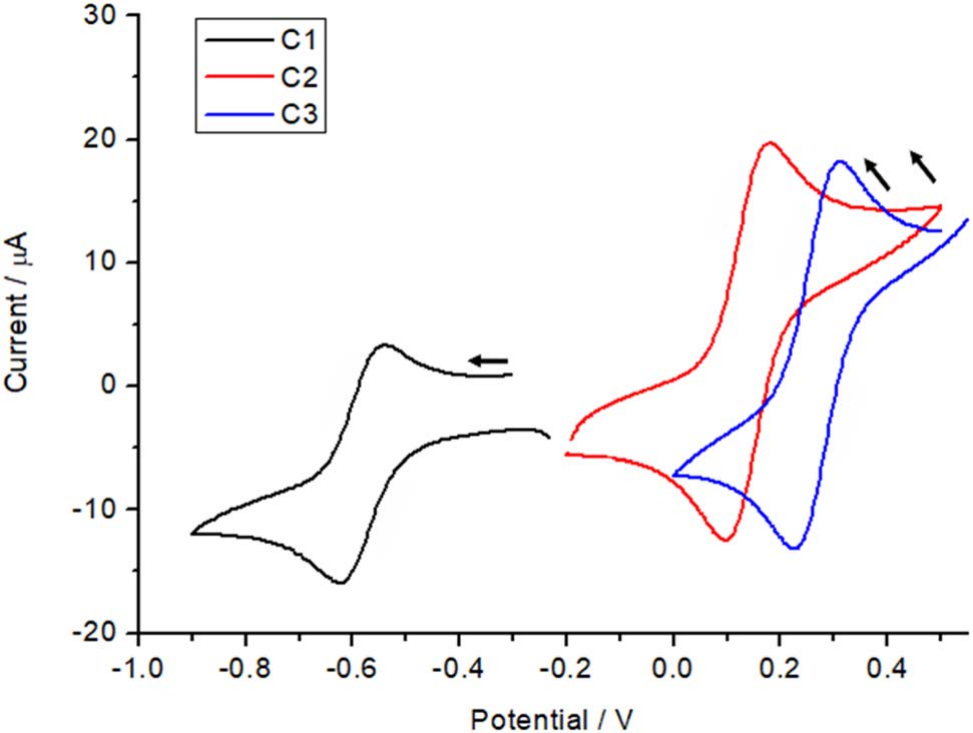
Cyclic voltammogram comparison of redox process from catechol for complexes C1–C3 in 1.0 mM with 0.1 Mn–Bu_4_PF_6_ in acetonitrile obtained with a scan rate of 100 mV s^−1^.

Cyclic voltammetry measurements were carried out on all three complexes C1–C3. The CV experiments were conducted using a three-electrode system with a glassy carbon working electrode, an Ag/AgNO_3_ reference electrode, and a platinum counter electrode. The electrolyte solution consisted of tetrabutylammonium hexafluorophosphate as electrolyte (0.1 M) and the complexes, C1–C3, (1 mM) in dried acetonitrile. The solution was purged with argon for 30 minutes prior to the measurement. The cyclic voltammetry measurements were carried out to investigate the effect of the ligand substituent on the catechol on the redox potential of the complexes.^32,33^ This will help us to understand the ligand field tuning imparted by three different catechols on the complexes C1–C3. For the complex with 3,5-di-*tert*-butylcatechol (C3), the strong electron-donating effect of the *tert*-butyl groups increases the electron density on the π-system of the ligand. This raises the energy of the ligand's highest occupied molecular orbital (HOMO), making it closer in energy to the acceptor orbitals on the metal centre. As a result, the semiquinone form is stabilized at room temperature. The increased electron density also weakens the ligand field strength, reducing the splitting of the metal d orbitals. These combined effects stabilize the reduced forms (catecholate and semiquinone) and shift the redox potentials to more negative values, as observed for C3, with a redox potential around −0.6 V (Fig. 6).

In contrast, the tetrachlorocatechol and tetrabromocatechol complexes exhibit redox potentials shifted to more positive values due to the electron-withdrawing nature of the halogen substituents. These groups lower the HOMO energy level, increasing the HOMO–LUMO gap and enhancing the ligand field strength imparted by these ligands on the Co(iii) centre. The stronger ligand field stabilizes the oxidized states of the ligand, particularly the quinone form, making oxidation thermodynamically more favorable. In comparison between the two halogenated complexes, the tetrabromocatechol complex shows the most positive redox potentials cantered around 0.3 V due to bromine's stronger electron-withdrawing inductive effect compared to chlorine. The complex based on tetracholoro catechol have the redox potential centred around 0.1 V. The reduced electron density on the catechol ring increases the oxidation potential, reflecting the destabilization of the reduced forms relative to the oxidized forms, *i.e.* the halogen substituents in the catechol make it easier for the complex to lose electrons, reflecting the greater favourability of oxidation relative to reduction.^32,33^

It is important to note that, these redox events are predominantly ligand-based rather than metal-centered. Thus, the direction of the redox potential shifts corresponds to the relative stabilization or destabilization of the reduced *versus* oxidized forms of the ligand, governed by the electronic effects of the substituents.

**Fig. 7:**
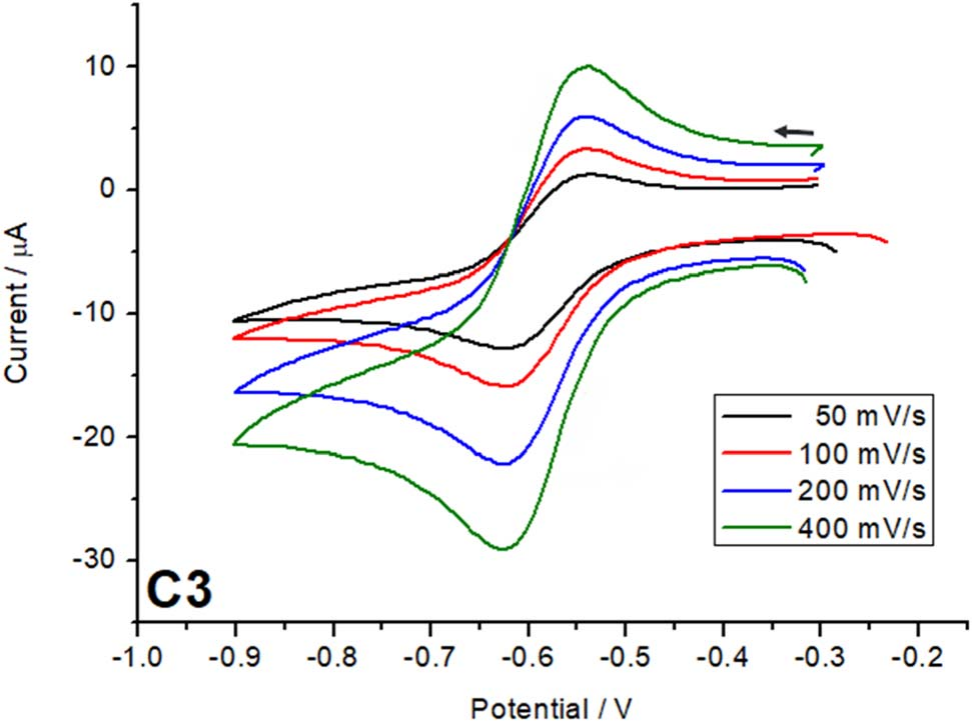
Scan rate studies of catecholate to semiquinolate redox process for complex C3.

Scan rate studies of the catecholate-based peak were performed (Fig. 7, S13 and 14†), which, from a clear linear increase in the current with increase in scan rate, indicate a fully reversible process. Other processes exhibited by the complexes (Fig. S12†) in the oxidation region can be assigned to the semiquinone, further getting oxidized to quinone around 0.9 V in the case of C1 and C2 and for all three cases the process around 1.1–1.3 V can be assigned to ligand L^sal^ based redox processes as known from the literature.^25,34^ The quinone is a very weak coordinating ligand and thus, once oxidized, might lead to decomposition of the complexes under the electrochemical conditions.

**Fig. 8:**
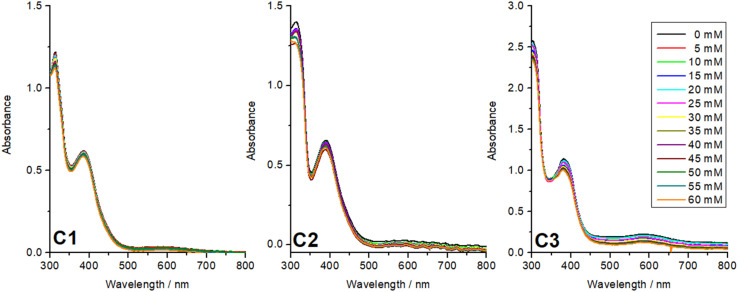
UV-vis acid stability test for complexes C1–C3 in the presence of 0–60 mM acetic acid in MeCN.

Finally, the complexes were tested for electrocatalytic green hydrogen production in acetonitrile with acetic acid as the proton source. The key advantages of using acetic acid as a proton source are (a) it is a weak acid and therefore allows prolonged catalytic activity, and (b) its polar nature reduces homoconjugation in MeCN.^35,36^ Before the catalytic testing all three complexes were tested with different equivalents of acetic acid by UV-vis spectroscopy. The UV-vis spectra recorded with 0–60 mM of acetic acid with 1 mM of the catalyst from 290–800 nm clearly indicate no significant shift or decrease in the intensity of the peaks upon the addition of acetic acid (Fig. 8). It is important to note that the total volume of the solution was kept constant. This indicates that the complexes are stable in the presence of acetic acid, and it can be used as a proton source for the electrocatalytic HER testing.

**Fig. 9:**
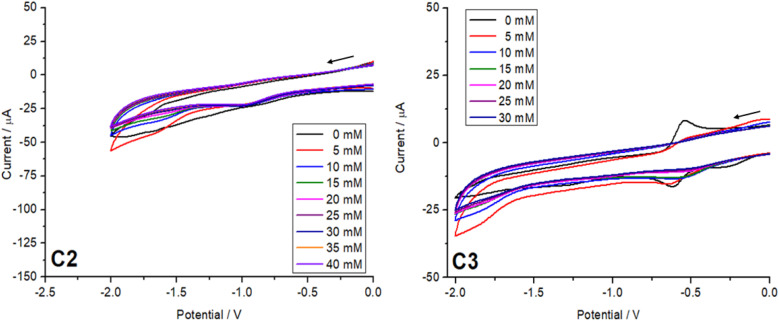
Cyclic voltammograms of C2 and C3 (1 mM) in MeCN at varying acetic acid concentrations (0–30/40 mM) at a scan rate of 100 mV s^−1^.

UV-vis spectroscopy confirmed the acid stability of the cobalt complexes C1–C3. However, when cyclic voltammograms were recorded for the reduction region with different equivalents of acetic acid for C2 and C3 no development of a catalytic peak was observed for both complexes. Despite the changes in acidity, no systematic increase in the catalytic current was detected (Fig. 9), and the expected electrochemical signature indicative of hydrogen production or other catalytic reactions emerged.^12,13^ This suggests that, under the tested conditions, both the Co(iii) complexes with 3,5-di-*tert*-butylcatechol (C3) and tetrabromocatechol (C2) demonstrate no noticeable catalytic activity. The poor catalytic performance observed is attributed to the highly stabilized Co(iii) oxidation state in these complexes, making it difficult to achieve the reduction potentials required for catalytically active Co(ii) or Co(i) species under the experimental conditions. Additionally, the fully saturated coordination sphere around the cobalt ion, combined with the low proton concentration further hinders efficient catalytic hydrogen evolution.

## 4. Conclusion

In conclusion, we successfully synthesized and characterized three cobalt salen ligand (L^sal^) complexes (C1–C3) featuring redox-active catechol ligands. Complexes C1 and C2 bear Co(iii) LS with *S* = 0 as evidenced by X-ray structural data analysis and magnetic data showing diamagnetic behaviour over the measured temperature.

In the case of the C3 complex, however, the redox-active 3,5-ditert-butylcatechol ligand formed a semiquinone, which was substantiated by SQUID magnetometry and EPR spectroscopy. CV measurements for all complexes revealed the effect of the substituents on the catechols in the redox potential. In the case of tetrahalogenated catechols the redox active catechol ligands stayed in the catecholate state because of the strong electron withdrawing nature of the halogen substituents, whereas in the case of complex C3, the electron donating substituents 3,5 ditertbutyl increase the electron density on the catechol, making it more susceptible to oxidation. Although preliminary electrocatalytic studies suggested only limited efficiency of these complexes as catalysts for hydrogen production under the conditions tested, their unique electronic and magnetic properties provide valuable insights for developing future redox-active molecular systems with tailored functionalities.

## Data availability

All the processed data are presented in the main manuscript and the ESI.† The crystallography data collected are deposited in the Cambridge crystal structure data base with the identification numbers 2415160–2415162.

## Conflicts of interest

There are no conflicts of interest to declare.

## Supplementary Material

RA-015-D5RA01958C-s001

RA-015-D5RA01958C-s002
