# Supplementary material for: Elucidating substituent effects in magnetic properties of redox active cobalt complexes and testing them as potential catalysts for HER
Source: RSC Adv. 2025 Jun 6;15(24):19287–94. doi: 10.1039/d5ra01958c (PMC12143256; doi:10.1039/d5ra01958c)
Supplement: RA-015-D5RA01958C-s001 [file RA-015-D5RA01958C-s001.pdf]

Supplementary Information (SI) for RSC Advances.
This journal is © The Royal Society of Chemistry 2025

Elucidating Substituent Effects in Magnetic Properties of Redox Active Cobalt Complexes and Testing them as Potential Catalysts for HER

Sriram Sundaresan, [a*] Julia Müller,[a] Luca M. Carrella,[a] and Eva Rentschler[a*]

[a]Department Chemie, Johannes-Gutenberg-Universität Mainz, Duesbergweg 10–14, 55128 Mainz, Germany. Email: rentschler@uni-mainz.de and ssundare@uni-mainz.de

S1. NMR Spectra: 2
S2. Infrared Spectroscopy: 3
S3. HRes Mass spectra: 6
S4. UV-Vis Spectroscopy 8
S5. Cyclic Voltammetry: 9
S6. Magnetic Data: 11
S7. Crystallography Information: 11
S8. Solid State EPR Spectra: 16
S9. ^{1}H-NMR of Complexes C1 and C2: 17

S1. NMR Spectra:

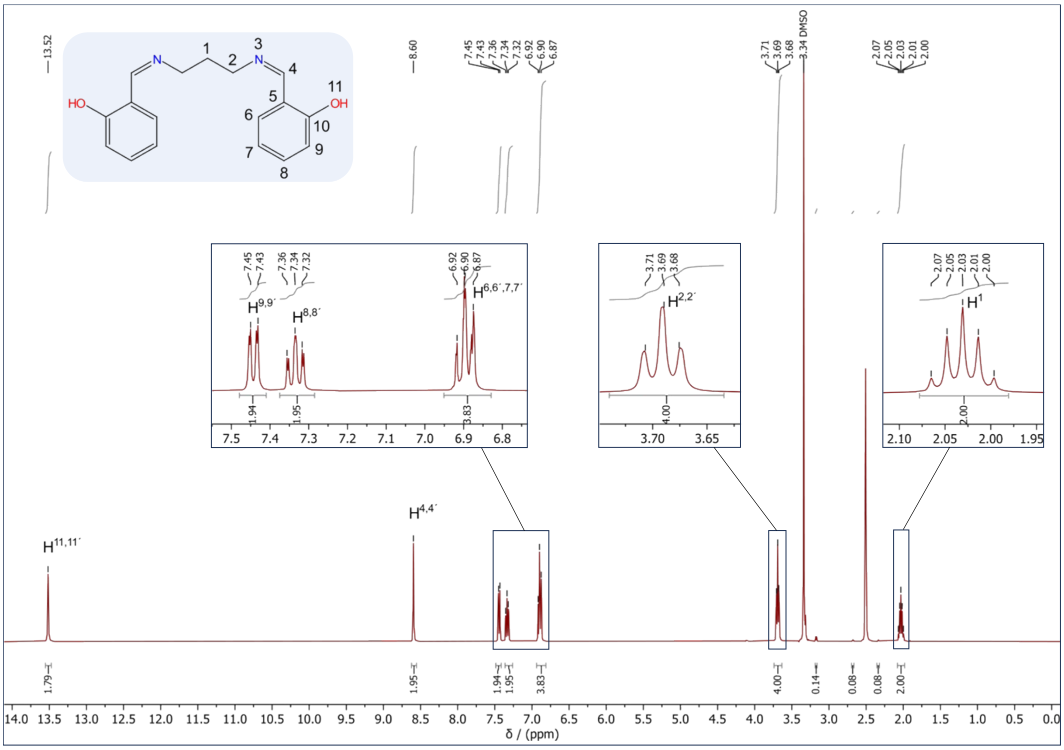

Figure S1: ^{1}H-NMR of N,N'-Bis(salicyliden)-1,3-propandiamin (L^{sal}) in DMSO [400 MHz].

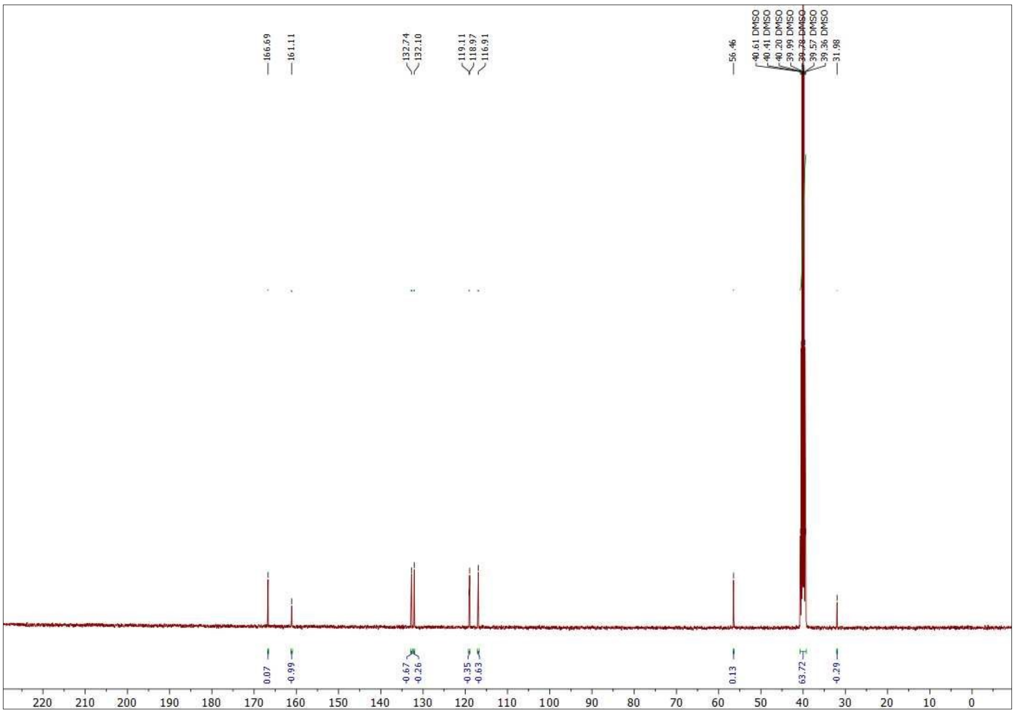

Figure S2: *^{13}C-NMR of N,N'-Bis(salicyliden)-1,3-propandiamin (**L^{sal}**) in DMSO [400 MHz].*

S2. Infrared Spectroscopy:

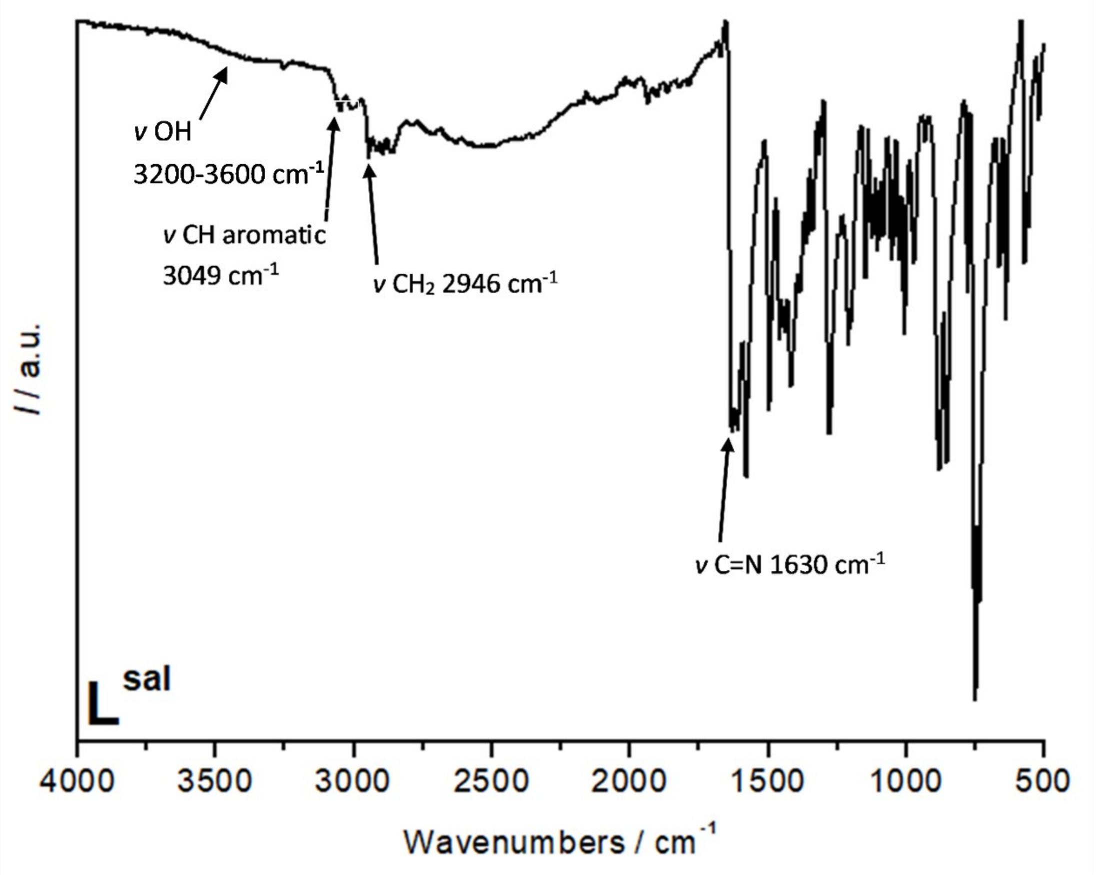

Figure S3: IR Spectrum of $\mathbf{L^{sal}}$.

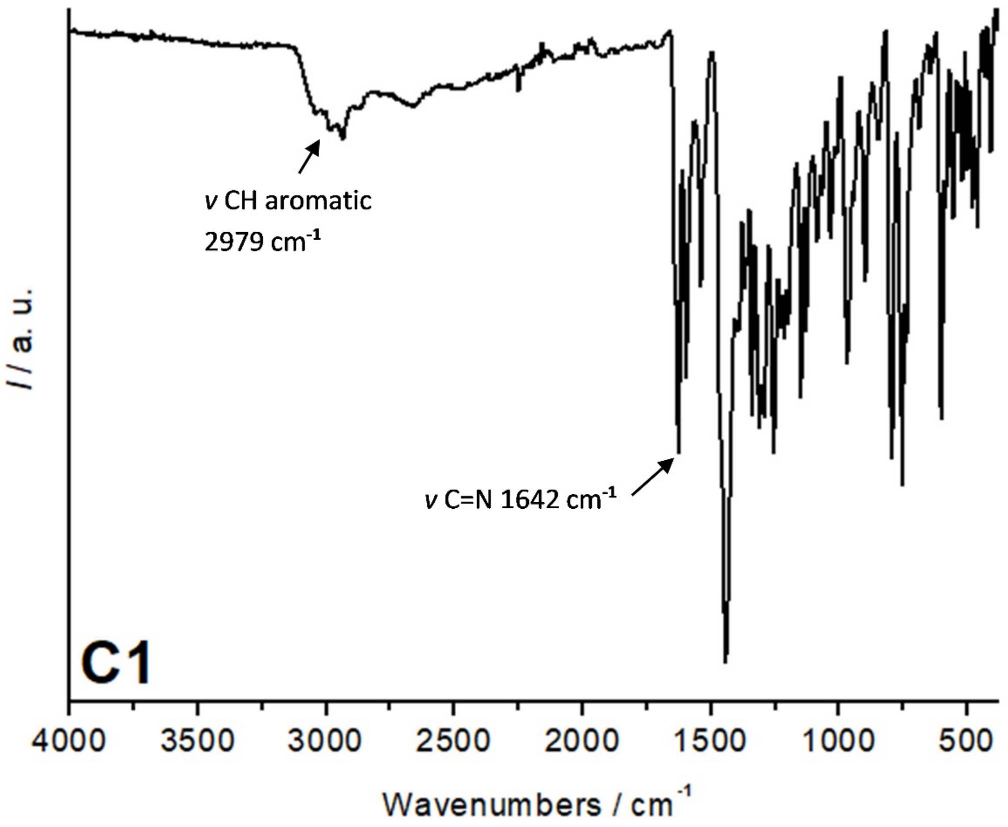

Figure S4: IR Spectrum of complex **C1** at room temperature.

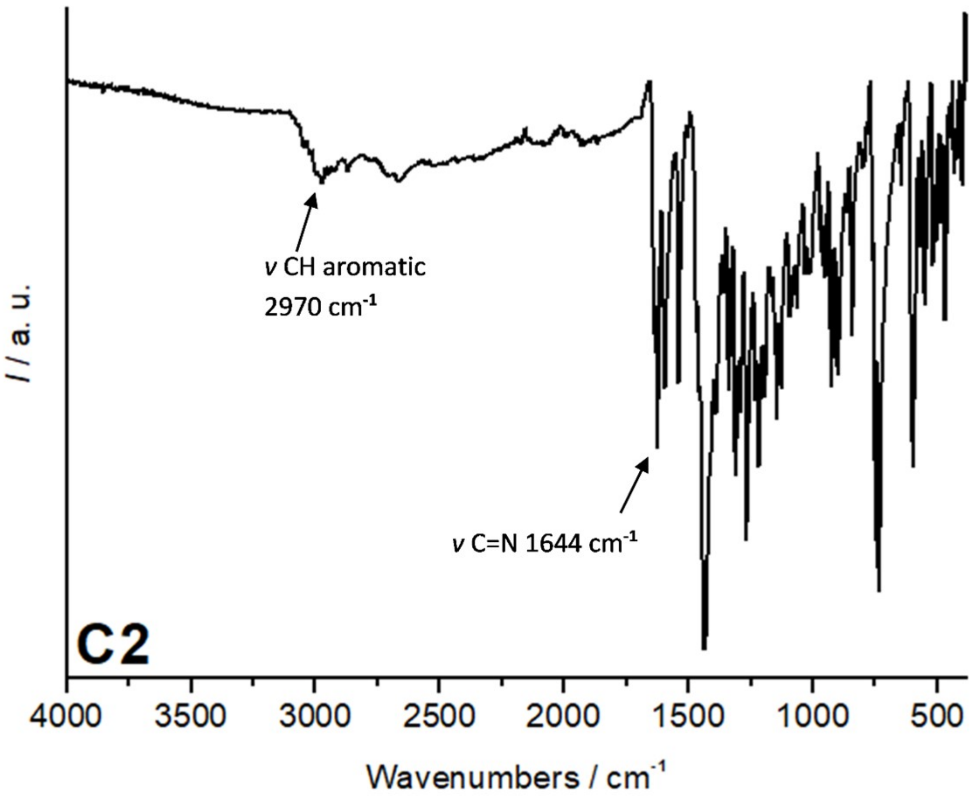

Figure S5: IR Spectrum of complex **C2** at room temperature.

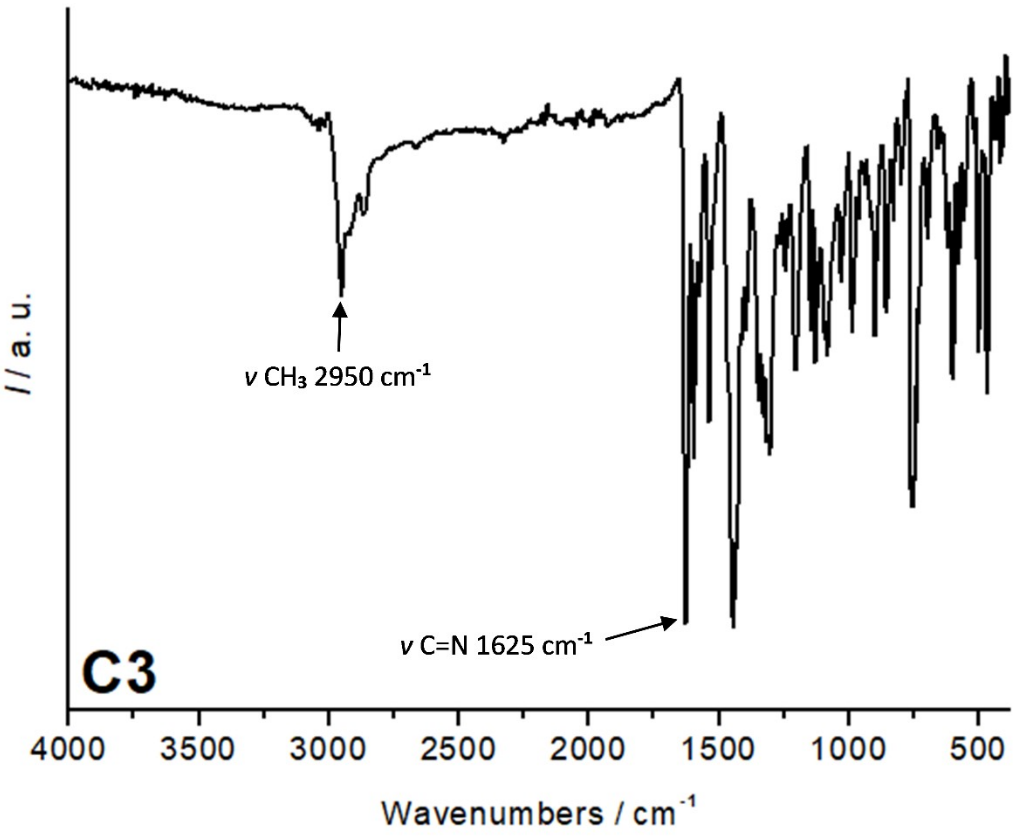

Figure S6: IR Spectrum of complex **C3** at room temperature.

S3. HRes Mass spectra:

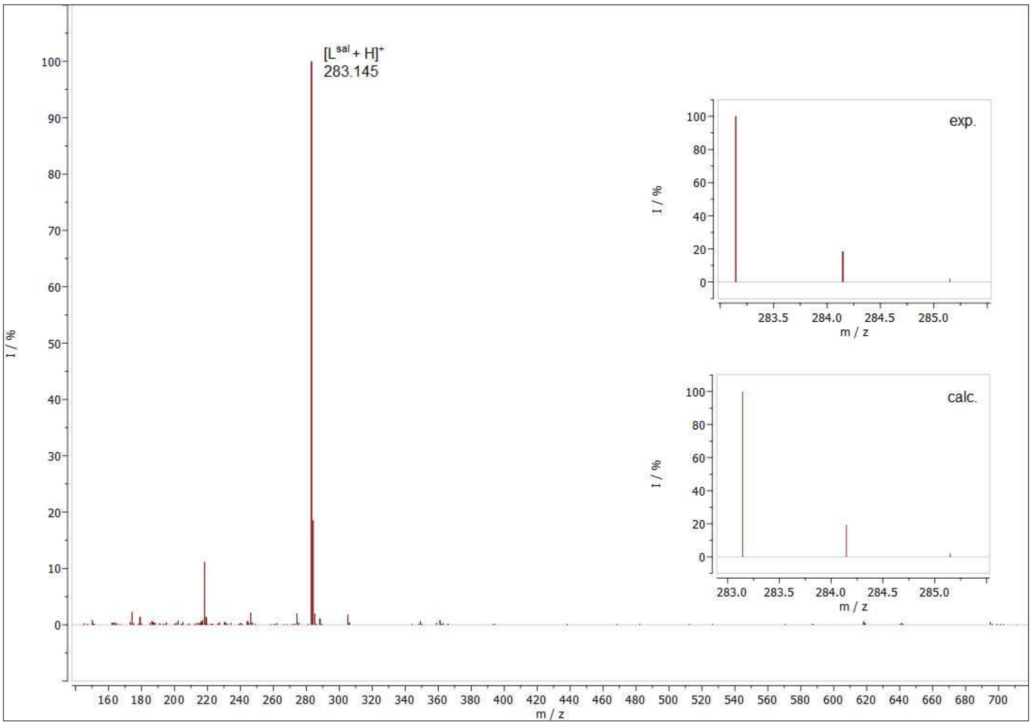

Figure S7: ESI (+) MS spectrum of $\mathbf{L^{sal}}$.

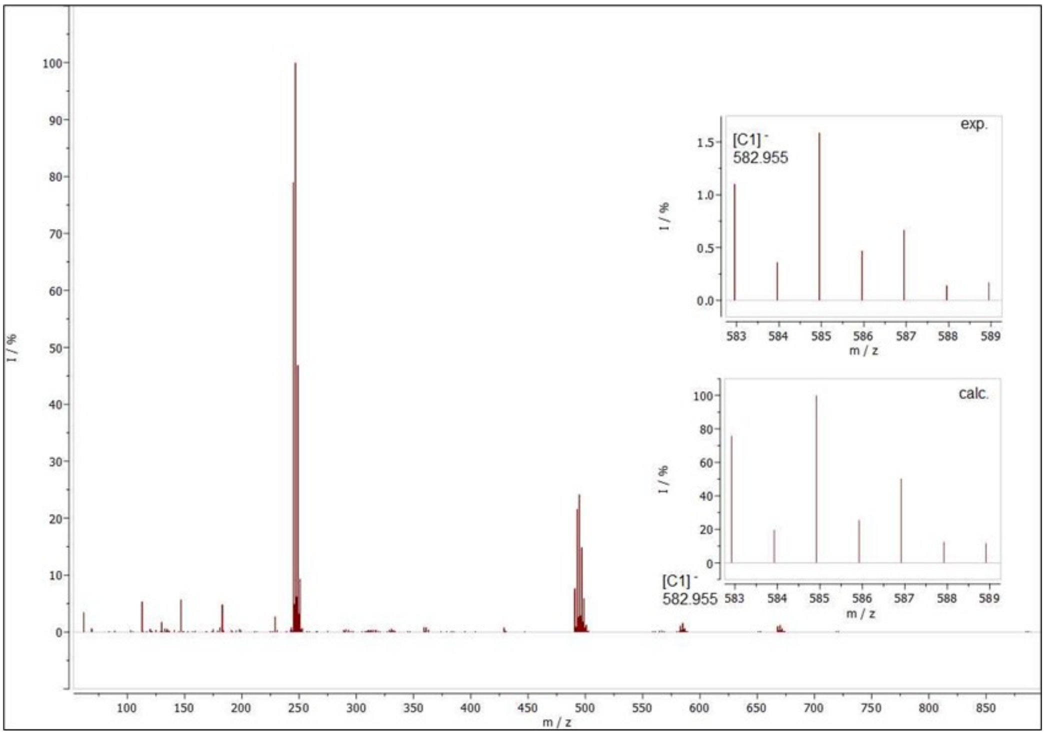

Figure S8: ESI (-)-MS spectrum of **C1**.

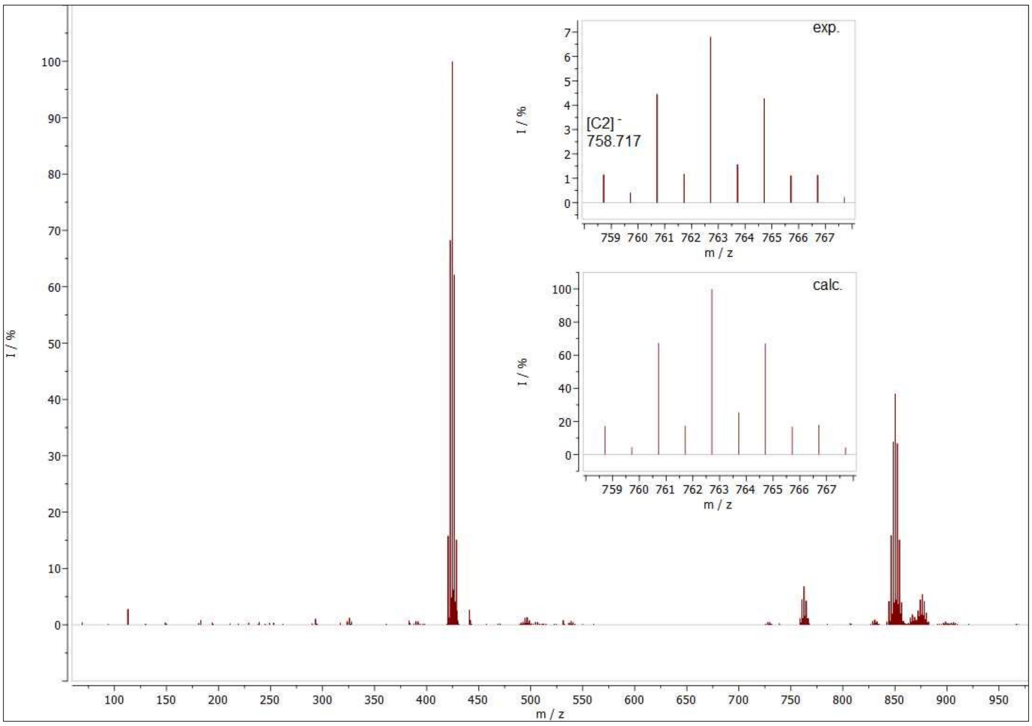

Figure S9: ESI (-)-MS spectrum of **C2**.

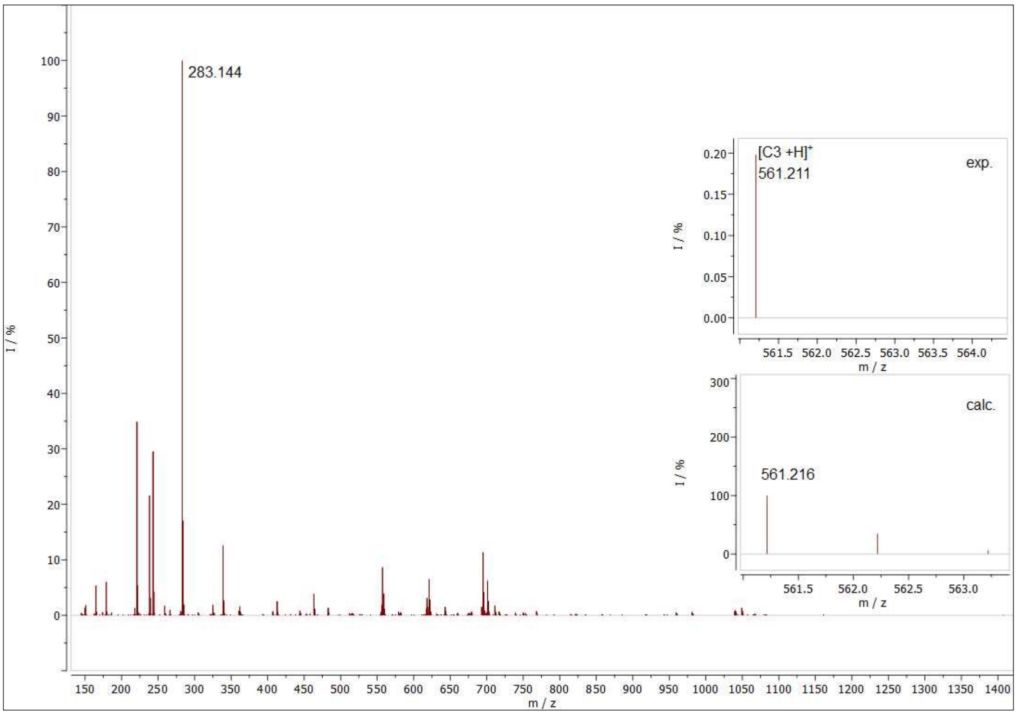

Figure S10: ESI (-)-MS spectrum of **C3**.

S4. UV-Vis Spectroscopy

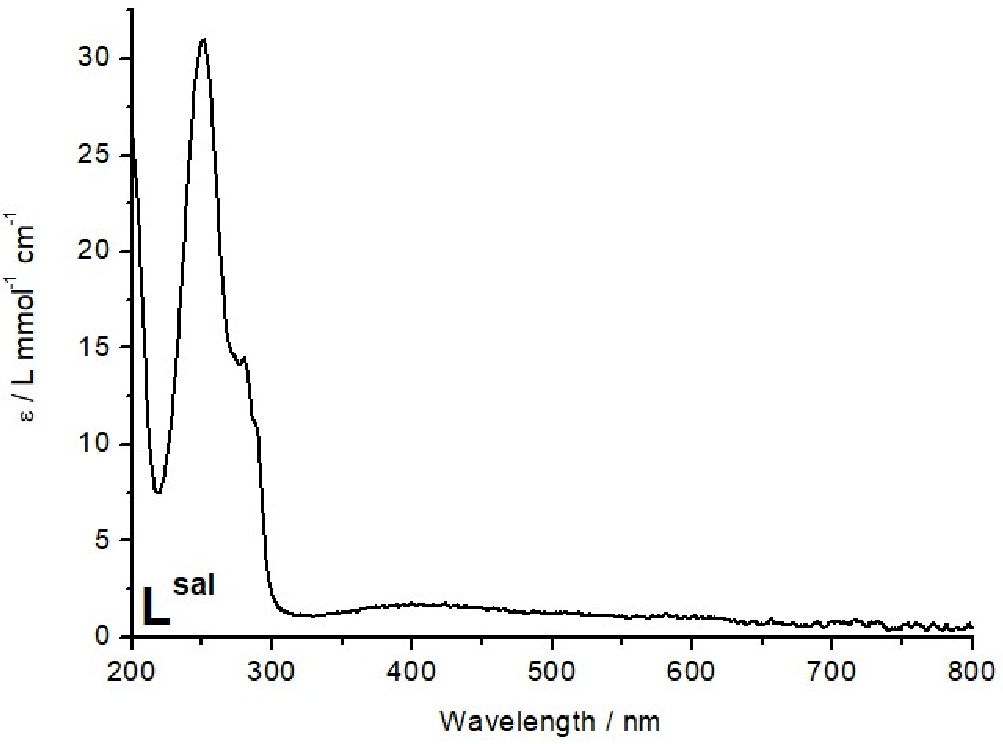

Figure S11: UV-Vis spectroscopy of the ligand **L^{Sal}**.

S5. Cyclic Voltammetry:

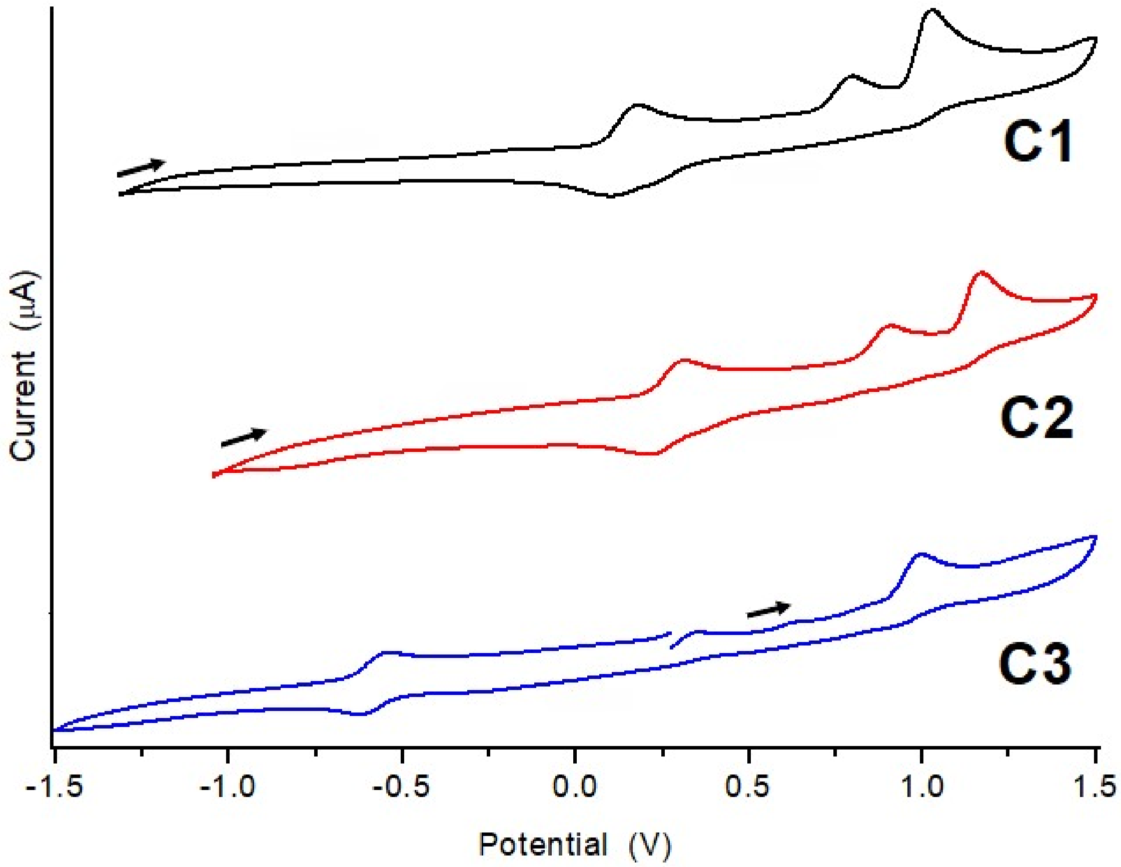

Figure S12: Cyclic Voltammetry of Complexes **C1-C3** in 1 mM acetonitrile at a scan rate of 100 mV/s.

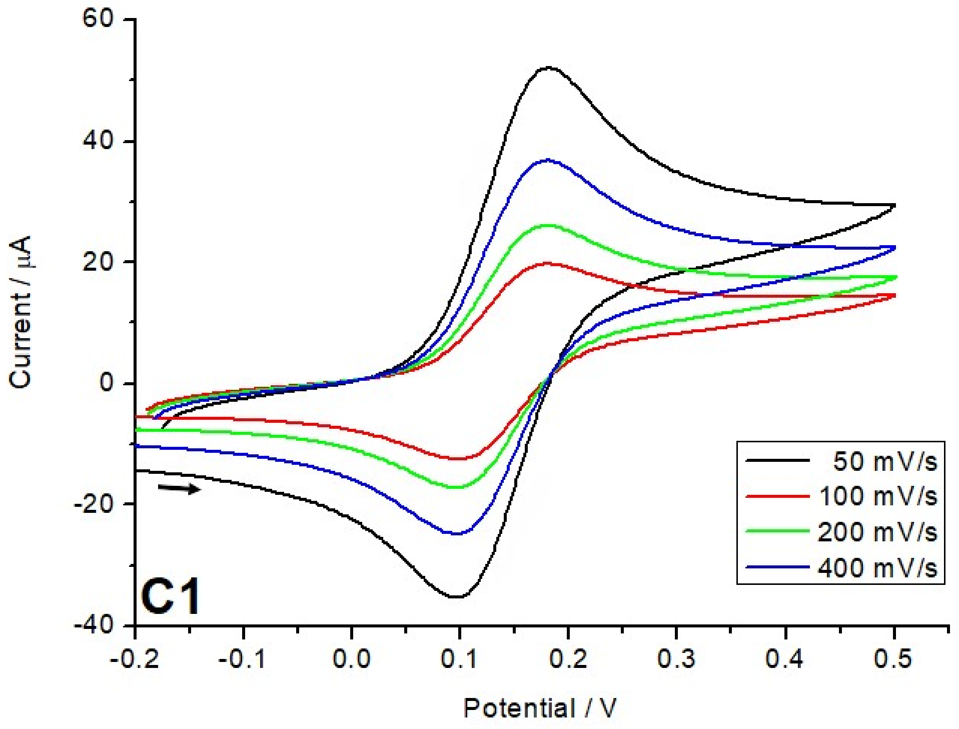

Figure S13: Scan rate studies of Complex **C1**.

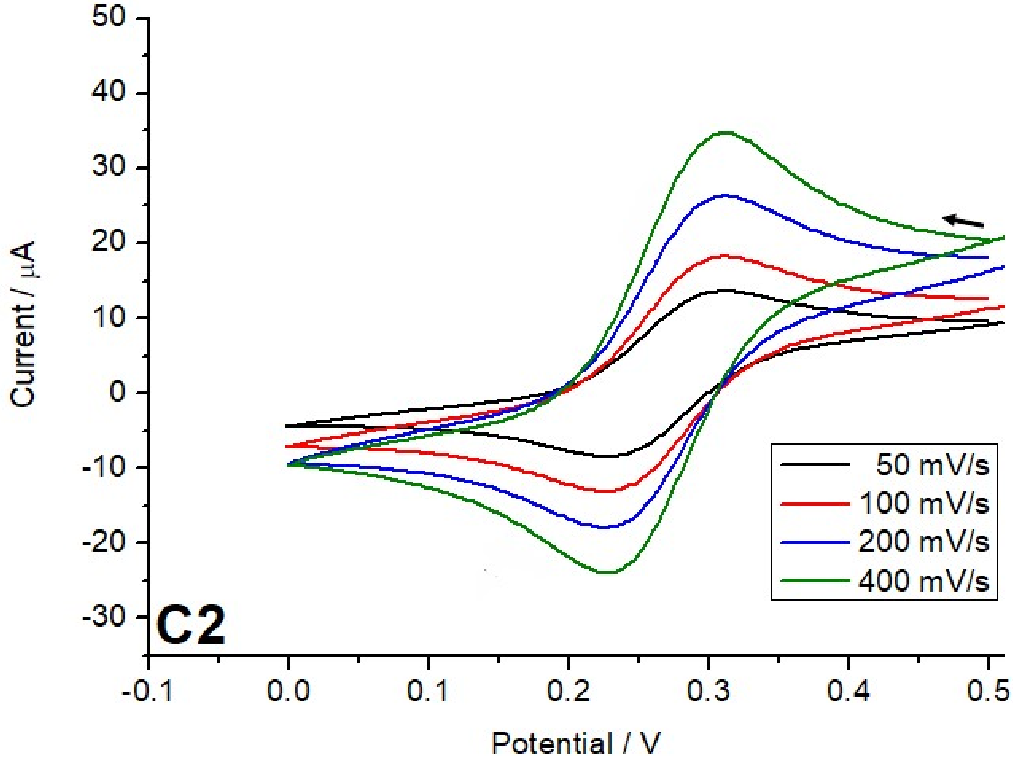

Figure S14: Scan rate studies of Complex **C2**.

S6. Magnetic Data:

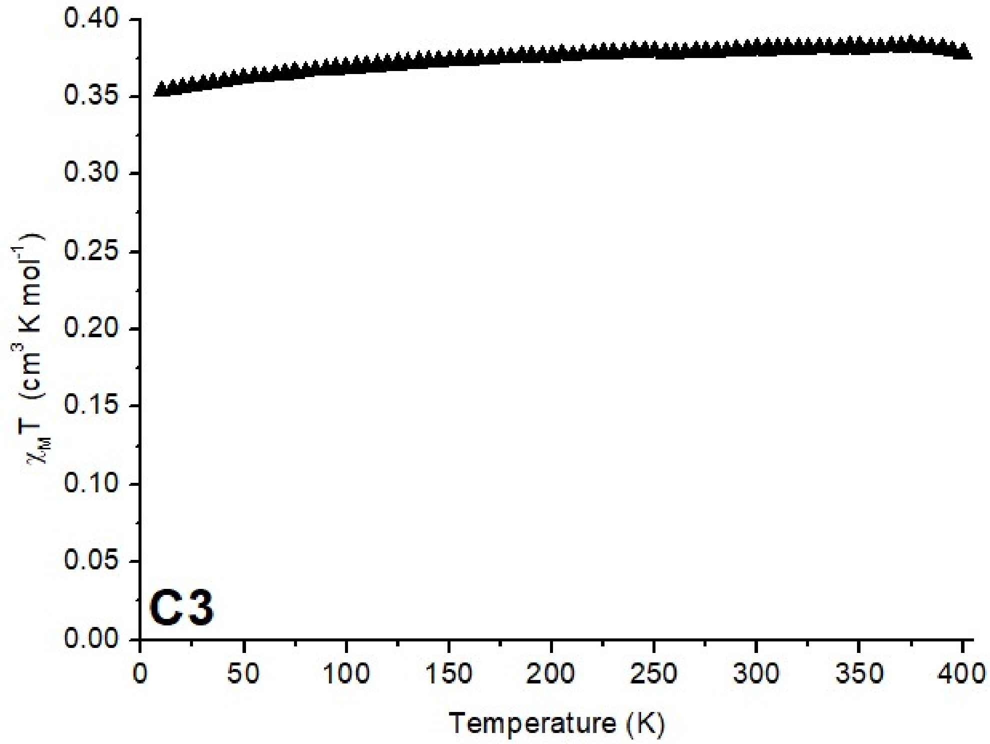

Figure S15: χ_MT *vs* T for complex **C3** from 10-400 K.

S7. Crystallography Information:

Table S1: X-ray Crystallography data for Complex **C1**.

Empirical Formula	$C_{29}H_{32}Cl_4CoN_3O_4$
Formula Weight / g mol^{-1}	687.3
Crysal Size / mm	0.65 x 0.357 x 0.2
Crystal system	Monoclinic
Space group	C2/c
Unit cell dimensions	
a / Å	21.235(5)
b/ Å	19.690(2)
c / Å	14.657(3)
α /˚	90
β /˚	97.922(15)
γ /˚	90
Volume / Å	6070.0(18)
Z	8
$\rho_{calc.}$ / g cm^{-1}	1.504
μ / mm^{-1}	0.957
F(000)	2832

Temperature / K	120
Diffractometer	STOE/IPDS/2T
Radiation	Mo-Kα
θ – range for data collection / °	2.500 < θ < 27.948
Index ranges	-27 < h < 27
	-24 < k < 25
	-19 < l < 17
Collected reflections	16349
Independent reflections	7216
Completeness	0.989
Max. and min. transmission	0.6601 and 0.3038
R_{int}	0.0314
R_{sigma}	0.0321
Data/ restraints/ parameters	7216/0/377
Goodness-of-fit on F^2	1.067
Final R_1 [I ≥ 2σ(I)]	0.0506
Final wR_2 [I ≥ 2σ(I)]	0.1189
Final R_1 [alldata]	0.0699
Final wR_2 [alldata]	0.1322

Table S2: X-ray Crystallography data for Complex **C2**.

Empirical Formula	$C_{29}H_{32}Br_4CoN_3O_4$
Formula Weight / g mol^{-1}	865.14
Crysal Size / mm	0.249 x 0.15 x 0.098
Crystal system	monoclinic
Space group	P2/n
Unit cell dimensions	
a / Å	14.9353(5)
b/ Å	9.62770(22)
c / Å	21.1281(8)
α /˚	90
β /˚	98.844(3)
γ /˚	90
Volume / Å	3001.93(16)
Z	4
$\rho_{calc.}$ / g cm^{-1}	1.914
μ / mm^{-1}	5.94
F(000)	1704
Temperature / K	120
Diffractometer	STOE STADIVARI

Radiation	Mo-Kα
θ – range for data collection / °	2.330 < θ < 32.753
Index ranges	-19 < h < 22
	-11 < k < 14
	-29 < l < 28
Collected reflections	33989
Independent reflections	9550
Completeness	0.86
Max. and min. transmission	0.1188 and 0.0583
R_{int}	0.0321
R_{sigma}	0.0351
Data/ restraints/ parameters	9550 / 0 / 373
Goodness-of-fit on F^2	1.072
Final R_1 [I ≥ 2σ(I)]	0.0439
Final wR_2 [I ≥ 2σ(I)]	0.1064
Final R_1 [alldata]	0.0591
Final wR_2 [alldata]	0.1153

Table S3: X-ray Crystallography data for Complex **C3**.

Empirical Formula	$C_{64}H_{78}Co_2N_4O_9$
Formula Weight / g mol^{-1}	1165.16
Crysal Size / mm	0.115 x 0.062 x 0.019
Crystal system	Triclinic
Space group	P-1
Unit cell dimensions	
a / Å	11.0407(6)
b/ Å	11.0875(6)
c / Å	24.7624(15)
α /°	92.046(5)
β /°	99.904(5)
γ /°	102.261(4)
Volume / Å	2909.7(3)
Z	2
$\rho_{calc.}$ / g cm^{-1}	1.33
μ / mm^{-1}	0.631
F(000)	1232
Temperature / K	120
Diffractometer	STOE STADIVARI
Radiation	Mo-Kα
θ – range for data collection / °	2.119 < θ < 26.000

Index ranges	-13 < h < 13
	-13 < k < 13
	-30 < l < 30
Collected reflections	33470
Independent reflections	11409
Completeness	0.998
Max. and min. transmission	0.9560 and 0.8433
R_{int}	0.0472
R_{sigma}	0.0815
Data/ restraints/ parameters	11409 / 0 / 726
Goodness-of-fit on F^2	1.042
Final R_1 [I ≥ 2σ(I)]	0.0541
Final wR_2 [I ≥ 2σ(I)]	0.1011
Final R_1 [alldata]	0.1007
Final wR_2 [alldata]	0.1181

Table S4: Metal donor bond length of complexes **C1**-**C3** at 120 K.

Metal-Donor	**Bond Length / Å (C1)**	**Bond Length / Å (C2)**	**Bond Length / Å (C3)**
Co-O1	1.929 (2)	1.923 (2)	1.884 (2)
Co-O2	1.897 (2)	1.896 (2)	1.894 (2)
Co-O3	1.914 (2)	1.910 (2)	1.929 (2)
Co-O4	1.891 (2)	1.890 (2)	1.912 (2)
Co-N1	1.906 (2)	1.906 (3)	1.912 (3)
Co-N2	1.917 (3)	1.910 (2)	1.917 (3)

Table S5: Bond length of dioxolane unit in **C3** at 120 K.

	**Bond Length / Å (C1)**
C18-C19	1.407 (4)
C19-C20	1.372 (4)
C20-C21	1.438 (4)
C21-C22	1.371 (4)
C22-C23	1.431 (4)
C23-C18	1.444 (4)

Table S6: Bond length of Imine in all three complexes **C1-C3**.

Complex	Bond length C=N / Å
**C1**	1.287 (3)

**C2**	1.287 (2)
**C3**	1.289 (2)

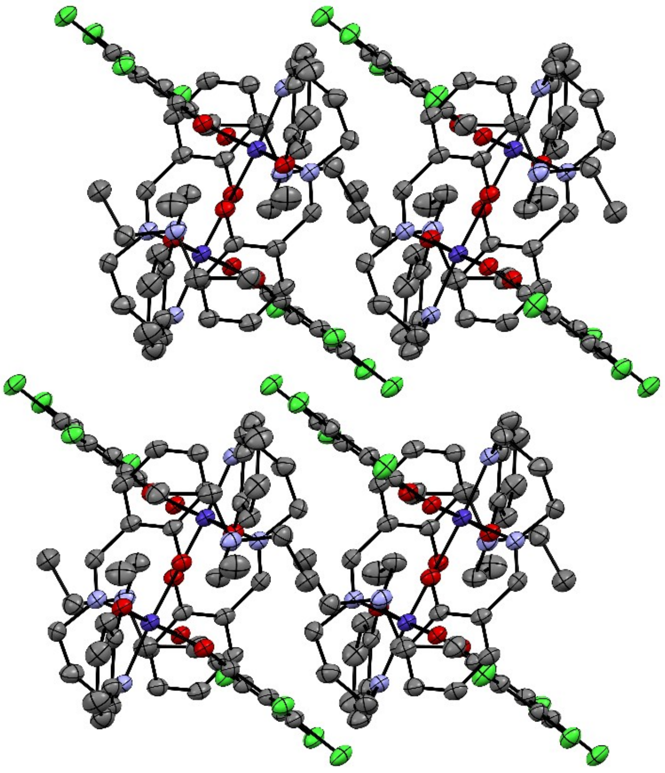

Figure S16: Packing diagram of Complex C1. Hydrogen atoms are omitted for clarity.

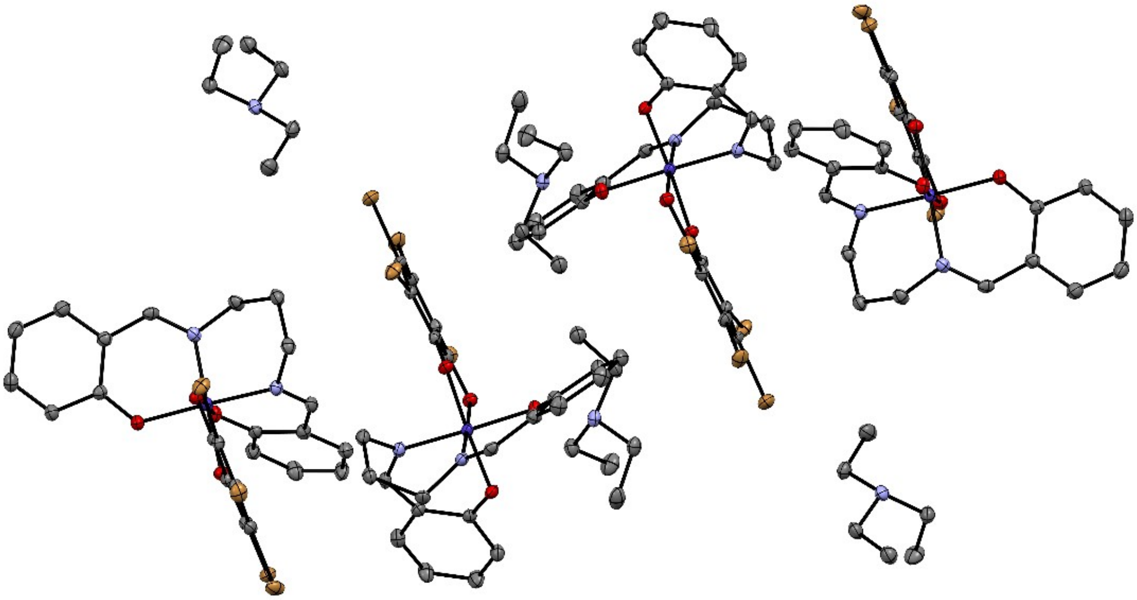

Figure S17: Packing diagram of Complex **C2**. Hydrogen atoms are omitted for clarity.

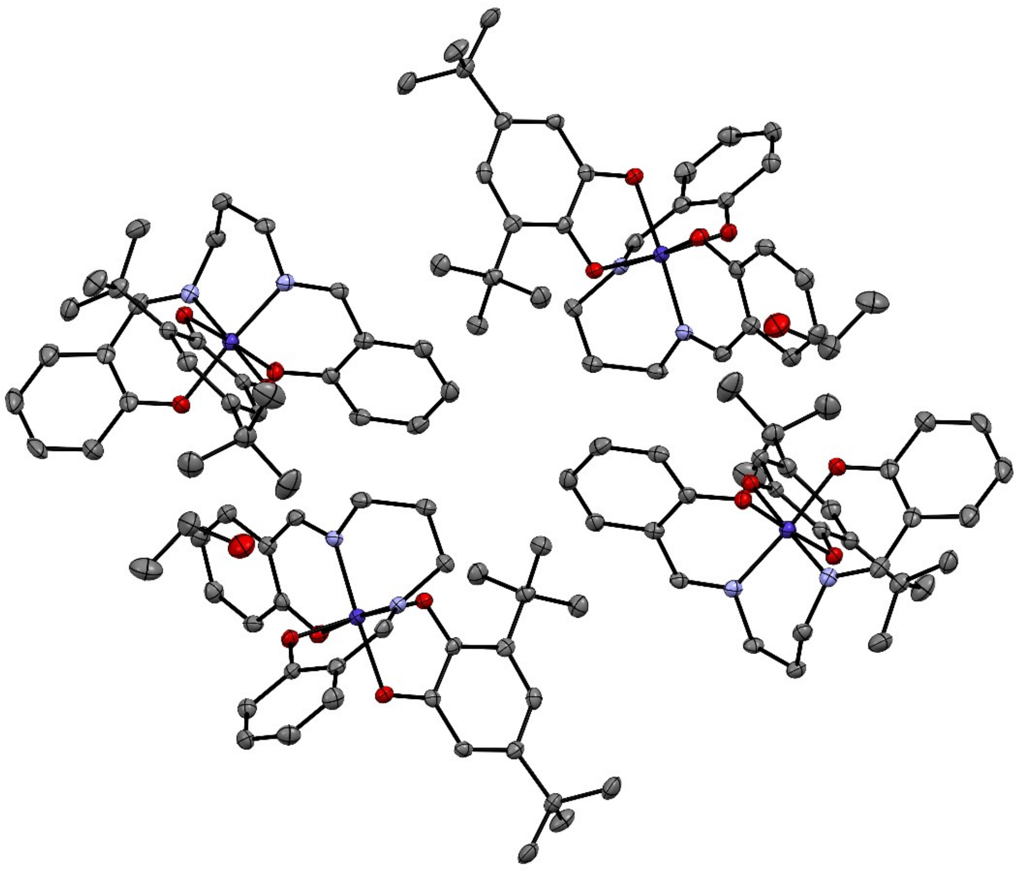

Figure S18: Packing diagram of Complex **C3**. Hydrogen atoms are omitted for clarity.

S8. Solid State EPR Spectra:

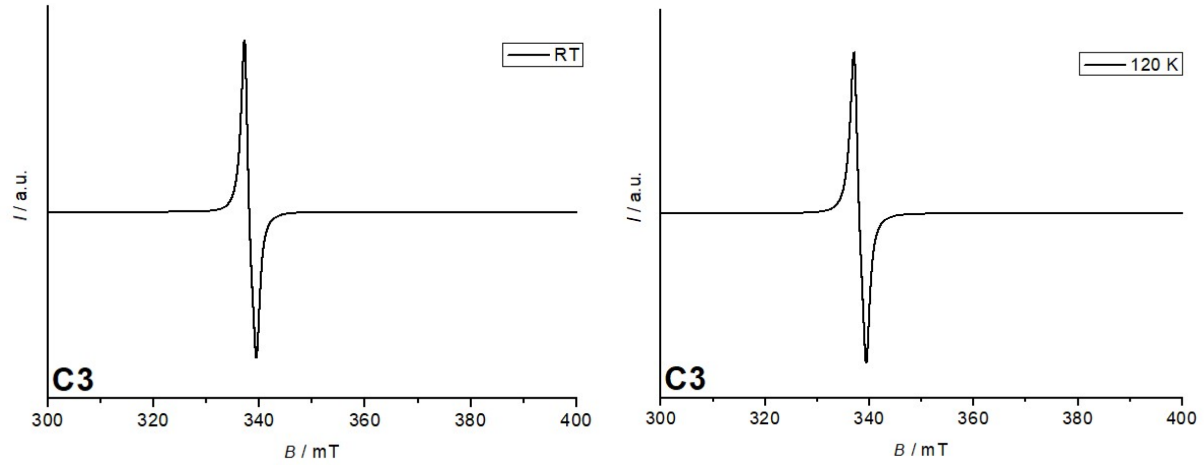

Figure S19: Solid State EPR spectra of complex **C3** at RT and at 120 K.

S9. ^{1}H-NMR of Complexes C1 and C2:

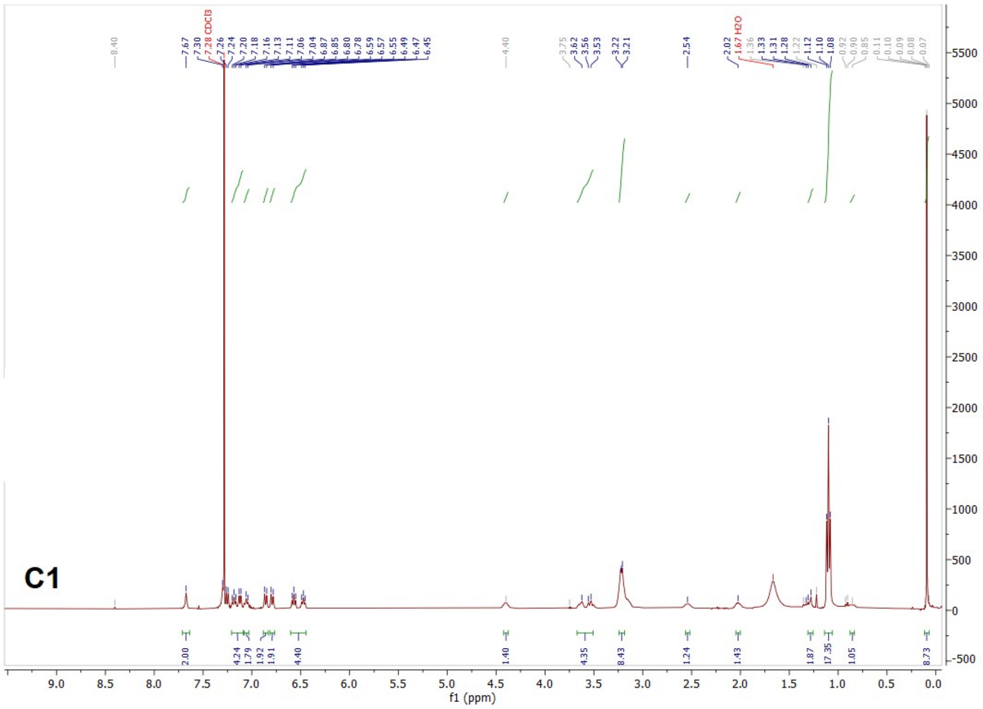

Figure S20: ^{1}H-NMR of Complexes **C1** in $CDCl_3$

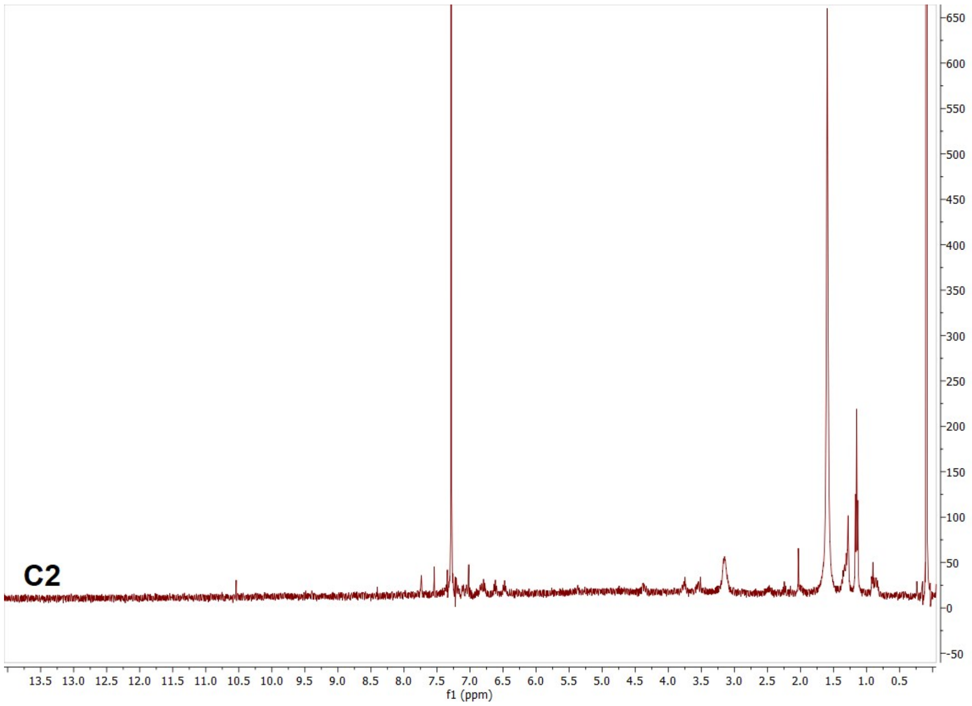

Figure S21: ^{1}H-NMR of Complexes **C2** in $CDCl_3$
